# Supplementary material for: Functional Analysis of NPC2 in Alarm Pheromone Recognition by the Red Imported Fire Ant, Solenopsis invicta (Formicidae: Solenopsis)
Source: Insects. 2025 Jul 25;16(8):766. doi: 10.3390/insects16080766 (PMC12386980; doi:10.3390/insects16080766)
Supplement: Supplementary file 1 [file insects-16-00766-s001.zip › Figure S1-S7.pdf]

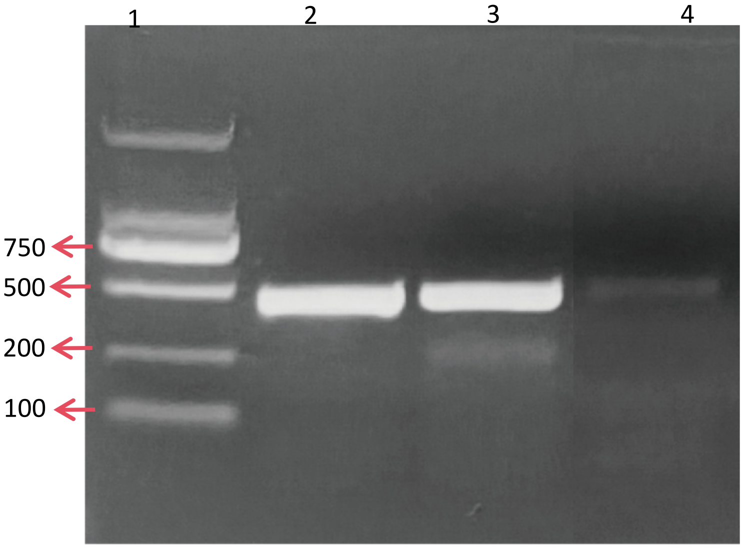

Figure S1. Banding patterns on a 1% agarose gel after multiplex PCR with Gp-9 allele-specific primers.Column 1, molecular weight markers expressed as base pairs (bp); column 2, PCR conducted with monogyne queen genomic DNA in the presence of primers 24bS, 25bAS, 26BS,and 16BAS; column 3, amplicon from lane 2 (Gp-9B allele-specific amplicon) digested with Hind.column 4, negative control.

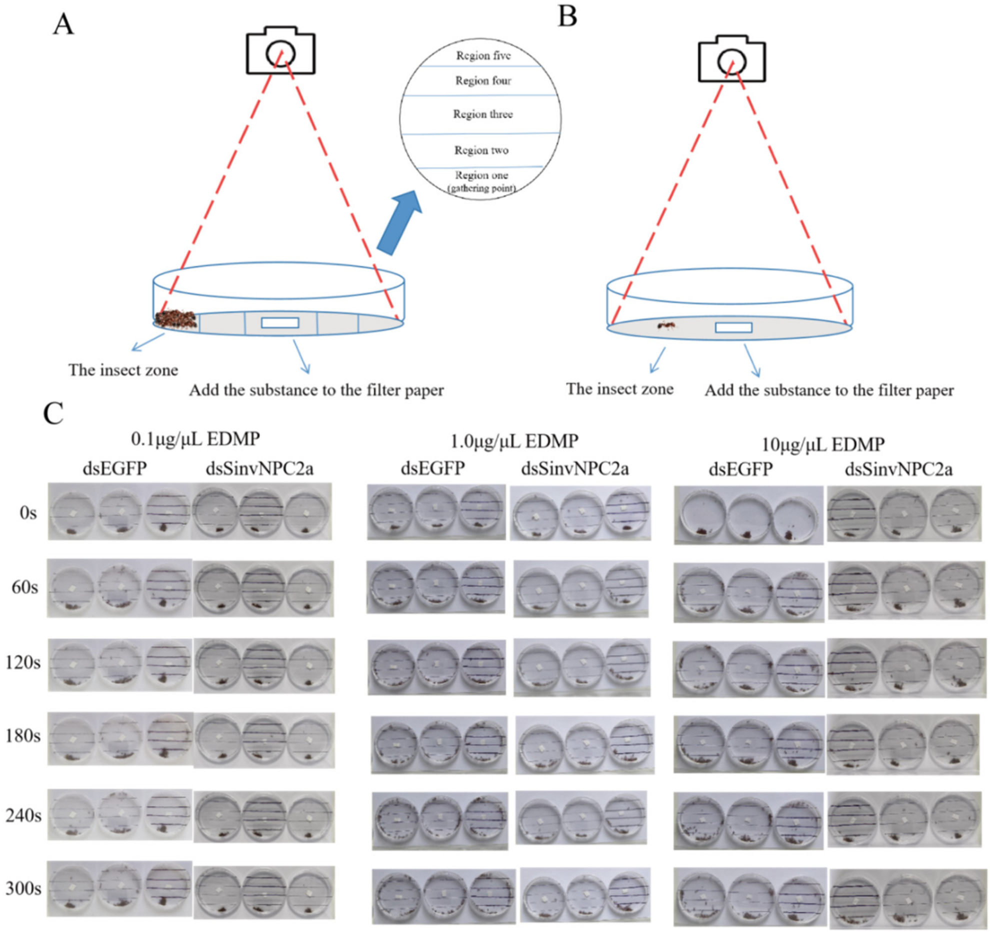

Figure S2. Red fire ant behavior measurement. (A) device Behavioral measurement device Region 1 is the aggregation point of red imported fire ants, and regions 2, 3, 4 and 5 are

diffusion areas. (B) A device that records the movement of a single red ant in an area within ten minutes. (C) The spread of worker ants to each region was recorded within five minutes.

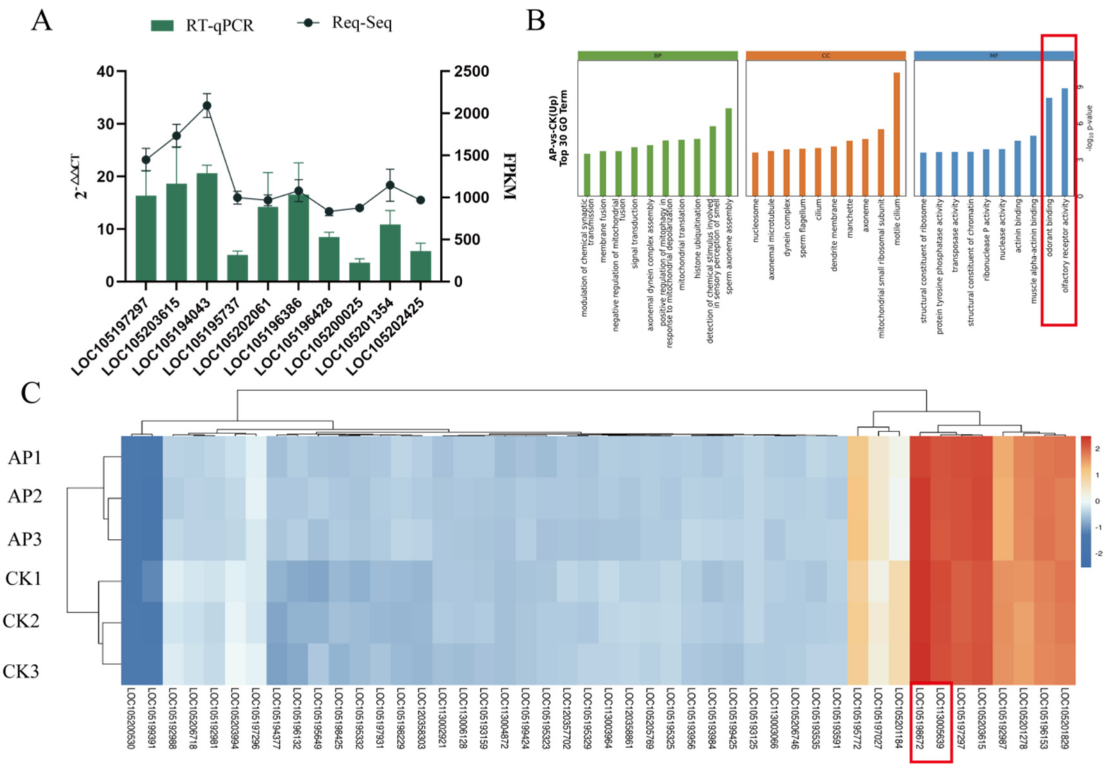

Figure S3. Analysis of transcriptome data and qPCR based validation. **(A)** Validation of qPCR ba sed on transcriptome FPKM values. **(B)** Functional classification of differentially expressed genes based on GO enrichment analysis; **(C)** Cluster analysis of differentially expressed genes. AP is E DMP treatment, CK is control.

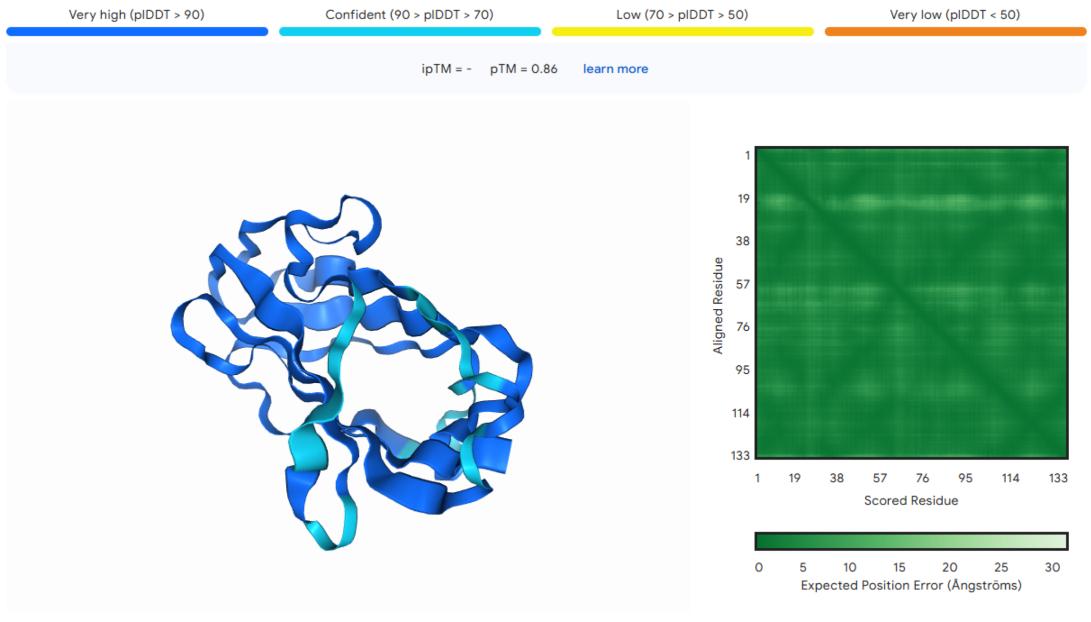

Figure S4. 3D model prediction and result analysis of SinvNPC2a.

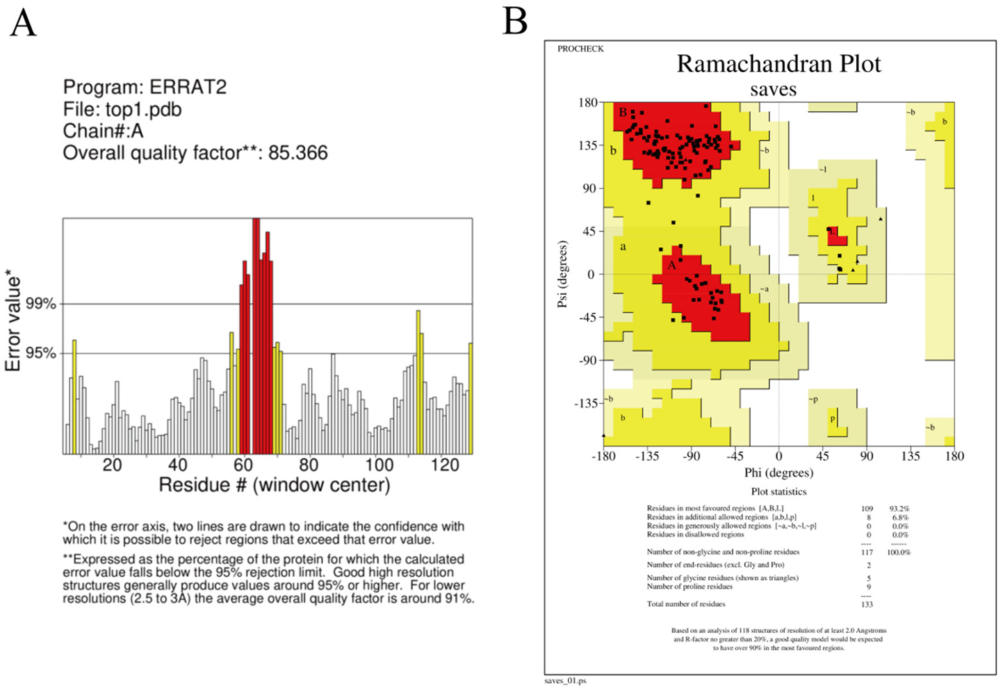

Figure S5. Quality assessment of SinvNPC2a model. (A) ERRAT evaluation results of 3D model of SinvNPC2a; (B) SinvNPC2a 3d model Lagrange diagram.

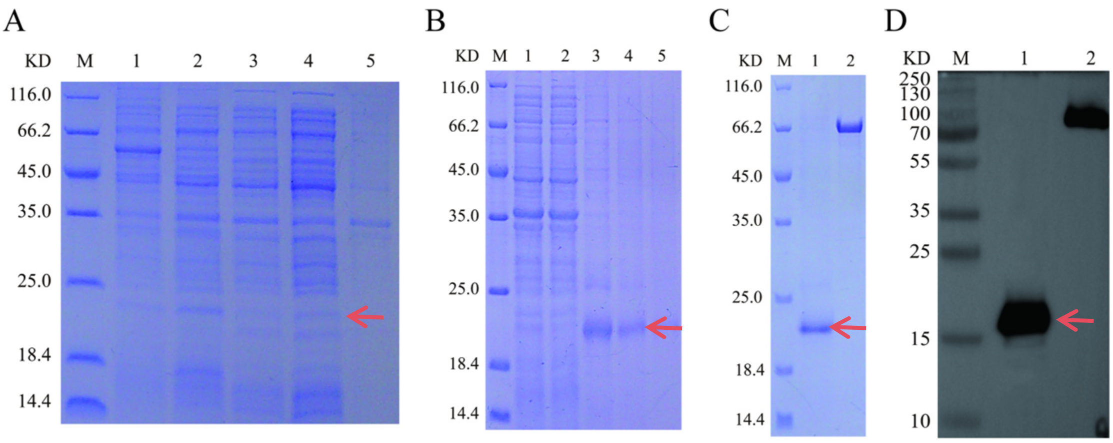

Figure S6. Expression and purification of SinvNPC2a. (A) SDS-PAGE analysis of protein expression identification; (B) SDS-PAGE analysis of protein purification; (C) protein detection analysis; (D) Western blotting analysis of protein identification.

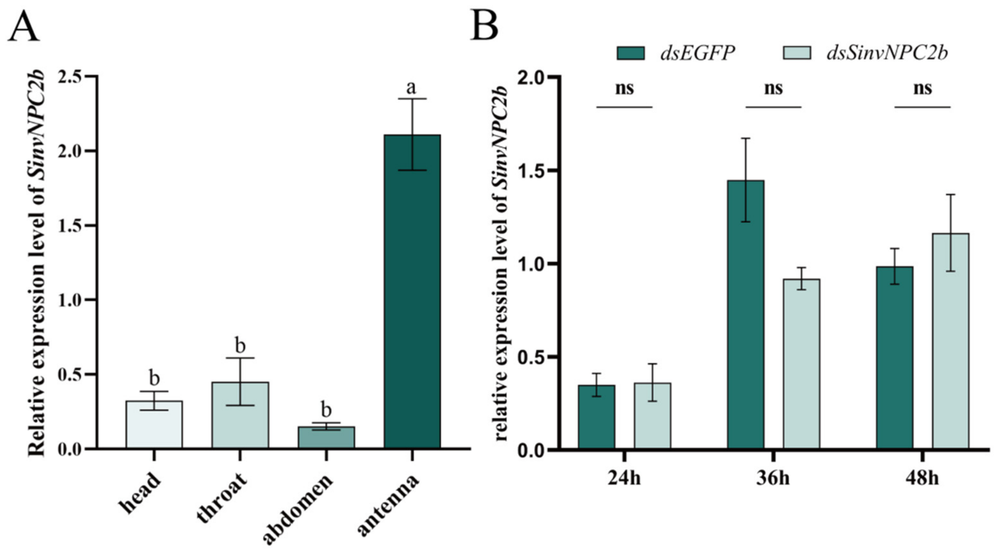

Figure S7. (A) The tissue-specific expression profile of SinvNPC2b. The mRNA levels of SinvNPC2b in different tissues were analyzed using qPCR. In the qPCR analysis, mRNA levels were normalized to ef1-β levels, with data from three biological replicates, each including four technical replicates. The bars and error bars represent the mean ± standard deviation (n = 4). Different letters on the bars indicate significant differences between tissues, as determined by Tukey HSD test (one-way ANOVA, $P < 0.05$).

(B) The interference efficiency of SinvNPC2b after feeding with gene-specific dsRNA. Expression of SinvNPC2b after feeding with dsSinvNPC2b. The expression levels of SinvNPC2s transcripts were analyzed using qPCR. The data shown are the mean ± SD from three biological replicates, with asterisks indicating significant differences detected by two-tailed t-tests (* P <0.05, ** P < 0.01, *** P < 0.001).
