# Supplementary material for: Functional Analysis of NPC2 in Alarm Pheromone Recognition by the Red Imported Fire Ant, Solenopsis invicta (Formicidae: Solenopsis)
Source: Insects. 2025 Jul 25;16(8):766. doi: 10.3390/insects16080766 (PMC12386980; doi:10.3390/insects16080766)
Supplement: Supplementary file 1 [file insects-16-00766-s001.zip › Table S1-S3 S5-S7.pdf]

Table S1. qPCR verification and analysis of gene and its specific primers

Number	Gene	Primer sequence	
1	SinvNPC2a	F:TTACAGCGCTTGCATTTGGC	R:CGAGACATCTTTGCTTGCTGA
2	SinvNPC2b	F:TGTCGATGGCAGCAAAAACG	R:CAACCAGTGAAACCCTGGGA
3	EF1-β	F:CCTGAAGACCGATAAGGGCAT	R:GATTGTTGTGTTGGTGGTTTCC

Table S2. T7 promoter-specific primers.

Number	Gene	Primer sequence	
1	dsSinvNPC2a	F:TAATACGACTCACTATAGGG TTACAGCGCTTGCATTTGGC	R:TAATACGACTCACTATAGGGCACCGGGAAT TTTACGCACAG
2	dsSinvNPC2b	F:TAATACGACTCACTATAGGG GTTCGAGGATGCTACGCAGA	R:TAATACGACTCACTATAGGGAGTGCGCTCA CTGCATTAGT
3	dsSinvEGFP	F:TAATACGACTCACTATAGGTA AACGGCCACAAGTTCAGC	R:TAATACGACTCACTATAGGGGCCATGATAT AGACGTTGTGGC

Table S3. Specific primers for PCR.

Number	Gene	Primer sequence	
1	SinvNPC2a	F: ATGACGCGAATCACTGTCTTTCC	R: TTATTGAATCTTCACCGGGAATTT
2	SinvNPC2b	F: ATGCTGAGGGAAACGATTCTCG	R: TCATTTTGTAATCTTCGATGGAAC

Table S5.NCBI Accession Numbers of All Amino Acid Sequences Used for Sequence Alignment.

Number	Gene	Accession Number
1	SinvNPC2a	XP_011161897.1
2	SinvNPC2b	XP_011170763.1
3	PdomNPC2	XP_015179114.1
4	CjapNPC2	BAO48214.1
5	MrotNPC2	XP_012149950.1
6	BimpNPC2	XP_024221484.1
7	AmelNPC2	XP_624310.2

Table S7. The docking results of SinvNPC2a and EDMP

Vina score	Cavity volume	Center (x,y,z)	Docking size (x,y,z)
-4.8	636	-1.0,6.0,2.0	17.0,17.0,24.0

Table S6. The GenBank accession/reference and amino acid sequence information for forty NPC2s used in the maximum likelihood tree.

NO.	Gene Name	Species	Amino acid squences	GenBank Accession/Renfernces
1	AaegNPC2	*Aedes aegypti*	MFKYLLLAALIPAVMLQNADNVDHESWPQVAIRPCAGVRPPP RGVRIEDCVELPCLLPRGRDANMAMDFTAIQDATNLNTVVTA TALGITAPYELPAERAAACNWLVQSRCPISAGEDLTYHLSMPV TAIYPLVSVTIEIDVVDQSQQSHGCFVVDTRVVAN	XP_001660218.1
2	AglaNPC2	*Anoplophora glabripennis*	MKVFLVLLGLAAYCSATQVKQCKGIGRSIEDLNERVKIGACNK PPCRLRKDSVVGITLKFKPDHDIKSLVNTVNANILGIPFPFAGV DGVDACKNIYNEDGTTKVGCNIKAGQEYLYKSEIEVLKIYPRV KTVVHWGLTEPDGQDVICFEVPARITN	XP_018560916.1
3	AmelNPC2	*Apis mellifera*	MYRMIVIILIVCLCFSPMYRAINIDDCGSKVGKLTSITLDCDMTK SVCDLIPDTNATIRIDFTLEKDVSKVNAIVHGIVMDIPIPFPLPNA DACQTPDSGITCPLNKGETYHYKNTLPVHKSYPKVSVTVKWQL KDENNEDIICVLIPARIK	XP_624310.2
4	BaffNPC2	*Bombus affinis*	MLRGIILVFVALLVVASATEVNQCGTGEKFEDSNQVKITGCDV PPCKLKRRTKAAVEQKFVPSEDVENVVNSVSAAIVGVPLPFIGV DGTSACDNIFKVDGTPAGCSLKKGVEYIYKREFPVLQIYPTISMV IHYALMDGNRTVACFEVPAKITNH	XP_050589977.1
5	BdioNPC2b	*Baryscapus dioryctriae*	MLRLLAILVALCAVVRAVEFRDCGSTAGQFDKVEVSDCDLGA DSCILKKGSDASIGLHFTPNKATTRVTAVVHGIIDDIDVPFPIPN AEACFDTHSGVTCPLPAGTGTSYHATLPVLAKYPTVSVKVKWE LKDEKGDNIVCVEIPVSLK	Functional Characterization of the Niemann−Pick C2 Protein BdioNPC2b in the Parasitic Wasp *Baryscapus dioryctriae* (Chalcidodea: Eulophidae)
6	BdorNPC2	*Bactrocera dorsalis*	MQQNFVFLLFAITVCSSVVYGLQFTDCGSKTGSFTKVIISDCDTT KNECILKRNTTASITINFALNEIASKITTVVHGKVMGVEMPFHL QNPDACVDSGLKCPLEKGETYEYKATLPVLKAYPKVNVLVKW ELQDQNSEDIICVQIPAKIQ	XP_011206785.1

NO.	Gene Name	Species	Amino acid squences	GenBank Accession/Renfernces
7	BimpNPC2	*Bombus impatiens*	MYRRIAIILVLCYLSFSPTCQALDIDDCGSKVGKFTSVTLDCDM<br>NKSVCDLIPNTNATINIDFTVDKDVSKVNAVVHGIVMDVPIPFP<br>LPNADACQAPDSGIKCPLKKGDATFHYKNTLLVLKSYPKVSVT<br>VKWQLKDENNEDIICILIPARIK	XP_024221484.1
8	BoboNPC2	*Bradysia odoriphaga*	MTGRIVVILIGLSVWIYGTNALEFTDCGSKVGKFTKVQVSDCVT<br>TSTACILKKNTNATISIDFELSTIDQCTKVTAVVHGVVMGIEMPF<br>NLPNADGCLNSGITCPIQKGSNYEYSTTLPVLKQYPKVKVDVK<br>WELIDENKDVIVCVMIPAKIQ	KAG4077358.1
9	ChisNPC2	*Cataglyphis hispanica*	MLRETALVFAALVVFAGATLVNHCDSEVPFEDSTQISVSECDK<br>PTCILKQGSTITIEIKLIPNRDIESLVNEVGAILFNVPLPFVGVDGT<br>NACDNIYNVDGSKAGCPLKKGVEYLYKNSFPILAFYPRVALVV<br>RYALREGNDKVVCFEVPAKITK	XP_050464070.1
10	CjapNPC2	*Camponotus japonicus*	MTRKDVGFSLLCVLCCIISSLAFVFEDCGSEVGKFSDIIISSCDPSE<br>EKCSIIRESEIHVSMKFTPSVDVKNVEAKAFGVLLDVPVPFPLKK<br>PEICKDPDSGVKCPLKKDVEIEYKVTFFVEKATPALSLEIMWEFR<br>NEKDEKITCVKFPAKIK	Niemann–Pick type C2 protein mediating chemical communication in the worker ant
11	DalbNPC2	*Drosophila albomicans*	MFKSYVLTLSLVLLLALMQLSGALEFHDCGSKTGKFTQVIIEGC<br>DTTKSECVLKRNTNVSISIDIALAEVASAVKTVVHGKVLGIEMP<br>FPLSNPDACQDSGLKCPLEKNETYRYTATLPVLKSYPKVSVLVK<br>WELQDQNNVDIVCVEIPARIQ	XP_034101125.1

NO.	Gene Name	Species	Amino acid squences	GenBank Accession/Renfernces
12	DlonNPC2a	*Diachasmimorpha longicaudata*	MFYHISLEHEILLHPRYFGPQLLDTVKQKLYTEVEGTCTGKYGF VVAVTTIDNIGAGIIQPGQGFVVYPVKYKAIVFRPFKGEVLDAIV TQVNKVGMFAEIGPLSCFISHHSIPADMQFCPNVSPPCYKSKEE DVVIQADDEIRLKIVGTRVDATGIFAIGTLMDDYLGLNCE	Identification and characterization of soluble binding proteins associated with host foraging in the parasitoid wasp
13	DlonNPC2b	*Diachasmimorpha longicaudata*	MAMETEDSSEKQLNNSGIDVDSGIENMEVEESDRKDTASRSRT TSSSIEVSPDQIHTVISRVLCVSWKDSTGHRIYLPQLSSALSSDVQ RSLHSTEIISQSLMEVLQTFTRDEDPLKGVTMETASDREDSPNSQ ASPLTSPVDSLPSYSLASLPLPPGEKGKKLEKSPQLKSLLYLLDCY TRVSVEERNQPKRSSVPPISDVLACLRAQCVQYSSLVLQGVLDT PDVQISTGYSPLLI	Identification and characterization of soluble binding proteins associated with host foraging in the parasitoid wasp
14	EmexNPC2	*Eufriesea mexicana*	MCRMIAAILILCYLSFSPCRALDIEDCGSKAGKFLSLNLDCDMT KAVCDLIPGTNATIRIDFTMEKDISKVTAVVYGIVMEMPIPFPLA NADACVTPDSGVTCPLKKGETYHYKNTLPVLKSYPKLSVTVK WQLIDENNEDIVCILIPARIK	XP_017763247.1
15	FvarNPC2	*Frieseomelitta varia*	MLRGIILVFVALLVVASATEVNYCGSSKTFEDPNSVKISGCDVPP CKLRRRTKASIEYKFTPDEDAENVVNNVSASIFGVPLPFVGVDG TTACDNIFNLDGTSAGCSLKKGVDYIYKREFPVLQIYPMISMTIH YALMDKNRTVACFEVPAKIIN	XP_043515907.1
16	HarmNPC2-1	*Habropoda laboriosa*	MLFFIIVTVLVASANAKYYTDCGSKLATVQKVEVSGCDAKNAE CVLRRNTNATFSIDFTPYKETKEVVTIVHGVIMNLPVPFPLPQP DACKDSGLTCPLGEEQSSSYHTTMPILKSYPRVKVDVKWELKD ENDEDLVCILIAAKIQ	Niemann-Pick C2 Proteins: A new function for an old family
17	HlabNPC2	*Habropoda laboriosa*	MLRGTIFVFAALIVIAAATEVNDCGSGAKFEDANRVRISGCSVP PCKLKRRTKASVEQKFVPDQDCDNIVNSVNAAVLGVPLPFVG VDGTSACENLFNADGTSAGCTLKKGVEYTYKREFPILQIYPTIS MVIHYELVQGNHTITCFEVPAKITN	XP_017788662.1

NO.	Gene Name	Species	Amino acid squences	GenBank Accession/Renfernces
18	HsalNPC2	*Harpegnathos saltator*	MLRETLFVFAAFAVFAGATIVKQCDSEELQLKDGVQVAVSDC DKPVCQLKQGTTVTIQIKFKPKKQIQSLTNAVSAIIFNVPLPFVG VDGTNACDNIYNADGSKAGCPLQEGVEYTYKNSFDVLAFYPK VSLTVQYALKEGDDQVLCFEIPSKITK	XP_011144363.2
19	LcupNPC2	*Lucilia cuprina*	MKQFVILFTLLAGFLQNSIYALQFTDCGSKIGKFTKIVVSDCDTT KNECILKRNSSVSITIDFSLAEDVTAVKTVVHGKVLGVEMPFHL QNPDACVDSGLKCPLEKDETYEYTATLPVLKAYPKVNVMVKW ELKDQNDEDIVCVEIPAKIQ	>XP_023305844.1
20	LhumNPC2	*Linepithema humile*	MSRIIVAFSLLCVLCCISSTLAFVFEDCGSEVGKFNEVSISSCDLSQ EKCSLIRGTEIRVSLKFTPSKDVAKIEARAFGVLLDVPVPFPLEKP ELCKDPDSNLKCPLKKDQEVEYKASFSVDKKVPALSVDVMWE FRNQDDEKILCVKFPAKVT	XP_012215343.2
21	LplaNPC2	*Harpegnathos saltator*	MTRKGIAFSPLCVLCCIVSSLAFVFEDCGSEVGKFNEISISSCDSSE EKCSIMRDSETHLSIKFIPSVDVSKIEAHAFGLLGDIPVPFPLEKP EVCKDSSSGINCPLKKDQEVEYKATFFVEKTTPALSLNVMWDF RNEKDEKIICVKFPVKIK	CAL1673194.1
22	MbicNPC2	*Melipona bicolor*	MLRGFILVFVALLVVASATEVNYCGSSKKFEDPNSVKISGCDVP PCKLRRRTKASIEYKFTPDEDAENIVNNVSASIFSVPLPFVGVDG TTACDNIFNLDGTSAGCSLKKGVDYIYKREFPVLQIYPTISMTIH YALMDRNRTVACFEVPAKIIN	KAK1137675.1
23	McinNPC2	*Macrocentrus cingulum*	MTSAKIFILWVLGVWVMIINGYEDCTNLKDISNPNGTSLGTLQ GFNVSDCDVNKVCIIDHNSTTTLSMDFTLNERVEVVNTKVEAS LGFISLELKIGHPAPCNDTNSGLECPLEPSDTAYHYTVTDQIKFR IRNQKINVKWSLLNEDAKTIVCAQVAVMFR	Molecular identification and functional analysis of Niemann-Pick type C2 protein in *Macrocentrus cingulum* Brischke (Hymenoptera: Braconidae)

NO.	Gene Name	Species	Amino acid squences	GenBank Accession/Renfernces
24	MdomNPC2	*Musca domestica*	MKQQFGILTIFIVAGLFLDNINALQFTDCGSKTGKFTKVSVSDC DTTKNECILKRNSTVSITIDFSLAEDVKAIQTVVHGKVMGVEMP FHLQNPDACVDSGLKCPLDKGETYEYTASLPVLKAYPKVNVM VKWELKDQNNEDIVCVEIPAKIQ	XP_005187474.1
25	MmedNPC2a	*Microplitis mediator*	MYRIILGLVLCLSVSTYAVIQDCGSKVGKYHNVTVSDCPSDAAT CILKRGTDATITISFDVDKEVSQVKAVVHGIIGGAPLPFPFSHPD GCQTSGLTCPLTKDNGPYQYSTSLHVEKLYPKLKVGVKWELKD ENEDTIVCALIPSEIK	Functional characterization of a Niemann–Pick type C2 protein in the parasitoid wasp *Microplitis mediator*
26	MmedNPC2b	*Microplitis mediator*	MLRNIFPVVLSLLCLTFLTEAVSYDQCKDAKDNHSIRLLTISNCE SAPCPLKRQTSVMVEEIFVAKKNVKSLTTSVFAKVAAFWLPFVS VHNKNACDNIYNMDNSIAGCPLKAGTEYKYRNEFPILSIYPTLT LPVQWAKDKNEIITCFQVYVKITN	Functional characterization of a Niemann–Pick type C2 protein in the parasitoid wasp *Microplitis mediator*
27	MphaNPC2	*Monomorium pharaonis*	MLRETSLVFAALVVFAGATVVYKCGSETPFEDPTQISISGCDKPE CPLKQGTTINVDIKLVPNREIKSLTNAVNAILFGVPLPFVGVDG TNACDNIYNPDGSKAGCPLKPNVEYLYKINFPVLSIYPRVALIV HYALKEGNEEIMCFEAPSKIIK	XP_012526691.1
28	MrotNPC2	*Megachile rotundata*	MYRMIAAVLSLYLCLTCRAFEIDDCGSKVGKFTSIKLDCDMAKS ECELIQNTNVTITIDFTVDKDVSKVTAIVHGIIMDVPIPFPLPNA DACADSTSGLTCPLAKGGPYKYKNMLAVHKSYPKLSVTVKWE LRDEDNETIICLLIPAKIK	XP_012149950.1
29	ObicNPC2	*Osmia bicornis*	MLRETILVFAALLVIASTTEVTKCGTGEKFEDSNQVKISGCDVPP CKLKRRTKYSIEEKFVPDQDVENLVNSVNAAILGVPLPFVGVD GTSACNSVFNADGSPAGCSLKKGVEYIYKQEFPILQIYPTVSMVI HYELMDGNHSVACFEVPAKITN	XP_029045758.2

NO.	Gene Name	Species	Amino acid squences	GenBank Accession/Renfernces
30	ObirNPC2	*Ooceraea biroi*	MLRETVLVFAALVVFAGATTVNQCGSEIPFEDSTQISVSDCDKP<br>TCILKQGTTVTIQIKLVPNRTINNLVNDVGAVLFNVPLPFVGVD<br>GTNACENIYNSDGSKAGCPLQPGVEYIYKNSFDVLPLYPRVSLI<br>VRYALREGNDKVICFDVPSKITK	XP_011352629.1
31	OtauNPC2	*Onthophagus taurus*	MSYKNGILIFMILCALFVKLNGEFSDCGSKMGKVDSISVTNCDA<br>DDDKCILRRNSNVTIQIQFTPQKDIKELKAIVHGVIMNAPIPFPL<br>PNGNGCVDSGVTCPLTSGTSYNYFTTLPVLKEYPPVSVDVKFEL<br>KDENDEDITCSMIPSKIK	XP_022919073.1
32	PdomNPC2	*Polistes dominula*	MTRSIFFCVFLVVSSILQLCYAVEFKDCGSKIGHFTKITISNCDTS<br>KTACELVRNSNVSLTIDFIPSQDISKIEAVIHGVIADVPIPFPLSHP<br>DVCTNPESNIQCPLKKDTEYTYKAVLPVENTYPKLSLKVRWEL<br>QDENKQDIICVSIPAKIK	XP_015179114.1
33	PexcNPC2	*Polistes exclamans*	MLRETLFVFATVVVILVSGTQVNHCSKNIPYEDANQVKISGCD<br>TPPCTLKKKNRISIEQTFVPEKDAEQLLTSVHATLLGIDLPFIGV<br>DGMNACDNIYDVDNKKVGCPVKKGTTYVYKTEFPILDIYPKV<br>NLVVYYALRNGNDIVSCFTVPAKITG	KAI4488086.1
34	PmexNPC2	*Polyergus mexicanus*	MTRKDVVFPLLCVLCCIVSSLAFVFEDCGSELGKFNEISISSCDLS<br>EERCSIIRGSEIHVSMKFTPSEDVSKIEALAFGVLLDVPVPFPLEK<br>PDVCKDSNSGINCPLKKDEGAEYKASFVVDKSTPALSLEVMWE<br>FRNEKDEKIICVKFPVKIK	XP_070160182.1
35	SinvNPC2a	*Solenopsis invicta*	MTRITVFPLLCVLCITSSFAFVFEDCGSEIGKMVELSISSCTDMTA<br>EKCVFTRGSDVNVNMKFSASKDVSEVTALAFGVMMEVPIPFPL<br>EKPKICSDPSSGVSCPLKKDQEYQYKSSFAVEKKTPPVSIEVMWE<br>FRSENDEKILCVKFPVKIQ	in this study

NO.	Gene Name	Species	Amino acid squences	GenBank Accession/Renfernces
36	SinvNPC2b	*Solenopsis invicta*	MLRETILVFAALVVLASTTVVNQCGSETPFEDATQISVSGCDVP ECQLKQGTKATIEIKLLPNREIKSVTNAVSALLFNVPLPFVGVD GTDACENIYNVDGSKNGCPLKPGTEYIYRNSFPILSLYPRVSLVV HYALREGNNELICFEVPSKITK	in this study
37	TcurNPC2	*Temnothorax curvispinosus*	MLRKTVLVFAALVVLTSATQVNYCDSETPFEDATQISVSGCDK PECLLKQGTDAVIEIKLSPNEDIQTLTNDVHAILFNVPLPFVGV DGTNACDNIFNADGSKAGCPLKKGVQYIYRNSFPVLSIYPRVSL TVRYALRQGNDRVICFEVPAKITK	XP_024887156.1
38	TnylNPC2	*Temnothorax nylanderi*	MSRIIVAFSLLCVLCCISSTLAFVFEDCGSEVGKFNEVSISSCDLSQ EKCSLIRGTEIRVSLKFTPSKDVAKIEARAFGVLLDVPVPFPLEKP ELCKDPDSNLKCPLKKDQEVEYKASFSVDKKVPALSVDVMWE FRNQDDEKILCVKFPAKVT	XP_071568956.1
39	WaurNPC2	*Wasmannia auropunctata*	MTRISVAFPLLCVLCSITFSFAFKFEDCGSEVGKLGEIVISNCDTS EDKCILTRGNEIRVSAKFTPSKDITDVTAYAYGVLLDVPVPFPLK KPDVCKDPDSGLNCPLKKDQEAEYKASFTLDKATPALSVDVL WEFKNENDEKLICLKFPAKVV	XP_011694636.1
40	XvioNPC2	*Xylocopa violacea*	MLRGTVLIFVALLAVANATEVNQCGTGETLEDSDRIKISGCDVP PCKLKRRTKATIEQKFVPDRDVQSLVNNVHAIVLGVPLPFVGV DGTSACENVFNADGTTAGCALKKGTEYTYKREFSVLQIYPTISM VIHYALMEGNNTIACFEVPAKITN	CAL7951227.1
